# Analyzing the impact of an MDG-Fund program on childhood malnutrition in Timor-Leste

**DOI:** 10.1186/s41043-024-00539-x

**Published:** 2024-04-04

**Authors:** L. Van der Spek, B. G. J. S. Sonneveld

**Affiliations:** 1 Athena Institute, VU University Amsterdam, De Boelelaan 1085, 1081 HV Amsterdam, The Netherlands; 2 Amsterdam Centre for World Food Studies, Athena Institute, VU University Amsterdam, De Boelelaan 1085, 1081 HV Amsterdam, The Netherlands

**Keywords:** Undernutrition, Food security, Small Island Developing State (SIDS), Under-fives, Program evaluation, Timor-Leste, Millennium Development Goals, Geographic information systems (GIS), Quasi-experimental

## Abstract

**Background:**

Timor-Leste’s food insecurity, propelled by political conflicts, a fragile economy and biophysical limitations that characterize mountainous Small Island Developing States (SIDS), is expressed in a high incidence (50%) of stunted children. Hence, the Millennium Development Goals Achievement Fund’s Joint Program’s (MDG-F JP) in 2009 was a timely intervention to reduce prevalence of underweight among under-fives. Since the impact of the program remains largely unclear, the current study investigates the contributions of the MDG-F JP on improving children’s nutritional status in Timor-Leste, in order to inform policymakers on how to make future programs more effective.

**Methods:**

Using bivariate analyses and multiple linear regression models we analyzed Demographic and Health Survey (DHS) data from under-fives in 2009–2010 and 2016, combined with spatially explicit data from geographic information systems (GIS). The analyses generated trends and factors associated with undernutrition, which were used in a quasi-experimental setting to compare districts that received the MDG-F JP with similar districts that did not receive MDG-F JP interventions.

**Results:**

A comprehensive set of factors dependent on seasons, locations, and individuals determine undernutrition in Timor-Leste. A positive impact of the MDG-F JP was found for the average severity of wasting, but not for stunting and underweight.

**Conclusions:**

The findings reinforce the pressing need for integrated and cross-sectoral programs, aimed especially at agricultural workers, mothers, and children. The agricultural challenge is to sustainably select, produce and conserve higher-yield and nutrient-rich crops, and educational enhancement should be aligned with local practices and research.

**Supplementary Information:**

The online version contains supplementary material available at 10.1186/s41043-024-00539-x.

## Introduction

In 2019, 21% of under-fives worldwide, 144 million children, experienced stunted growth, and an additional 47 million were underweight [69]. These figures are appalling, especially since it is well known that childhood undernutrition causes health problems throughout a person’s life, such as limited physical and cognitive development, cardiovascular diseases, and obesity, all of which have a direct effect on educational, labor and social capacities [4, 39]. Despite the focus of the Millennium Development Goals (MDGs) on undernutrition, the target to halve the global prevalence of underweight under-fives between 1990 and 2015 (MDG 1.8) was not achieved [73]. Low- and middle-income countries in particular were far from achieving this goal, mainly due to a lack of investment in effective child nutrition [2, 22, 63].

Among the lower middle-income countries, Timor-Leste (East Timor) is no exception; it failed to meet its target of reducing the prevalence of underweight among under-fives to 31%. Hence, Timor-Leste sadly remains the lower middle-income country with the second-highest child undernutrition rates [6, 77]. The stunting prevalence of 50% among under-fives is surpassed only by Burundi and Eritrea [21, 70]. Demographic and Health Surveys (DHS) and Living Standard Surveys over the last 20 years show that the percentage of stunted and underweight children in Timor-Leste has fallen slightly, though this trend is not statistically significant [31, 35, 54].

The alarming child undernutrition rates in Timor-Leste are to be understood and addressed within the Small Island Developing State’s (SIDS) unique socio-economic, political, institutional, cultural and biophysical context. The agricultural potential of Timor-Leste is restricted by its steep and stony slopes, poor soil suitability and trends of increased rainfall and temperatures due to climate change [5, 50, 54]. The small proportion of workable land and remoteness from major markets translate into high import costs for a limited range of products that are often high in calories, fat and sugar [25, 65]. Timor-Leste’s vulnerability is further characterized by devastating effects of its history of conflicts on educational, political, economic and social capital and infrastructure [27, 46, 54].

In this context, the Millennium Development Goals Achievement Fund’s Joint Program (MDG-F JP), entitled ‘Promoting Sustainable Food and Nutrition Security in Timor-Leste’, was set up. Nearly 90% of the MDG-F JP’s total budget of USD 3.5 million was allocated to improving health and nutrition status of the under-fives, as well as for pregnant and nursing women [44]. Yet, the MDG-F JP’s success was reported to be limited: it proved a challenge to implement sustainable changes that could be continued within the systems and partnerships without further support from MDG-F [44, 52]. In addition, the MDG-F JP’s impact on the nutritional status of Timor-Leste’s population remained largely unexplained because of the absence of baseline surveys, lack of appropriate sample designs and inadequate monitoring.

Overall, evaluations of food security programs were conducted with a relatively narrow scope, focused only on output results and impact, and so failing to gain a good understanding of the broad underlying and multifaceted set of determinants that determine the project’s failure or success [22, 45]. For this reason, the research aimed to investigate the contributions of the MDG-F Joint Program to children’s nutritional status in Timor-Leste. The study both compares anthropometric data over time, and also explains the prevalence of malnutrition in Timor-Leste in its dependence of demographic and household characteristics, as well as the prevailing natural endowments. The findings of this causality analysis are used for a conditional comparison between areas with and without the MDG-JF programs.

The major scientific challenge of this study was to attribute and isolate the impact of the program to changes in in the under-fives’ nutritional status among the many confounding factors that influence this process. We therefore formulated the following research question: How did the MDG-F JP improve the nutritional status of under-fives in Timor-Leste? And, what lessons can be learned about context-specific determinants of food security? The remainder of this paper is structured as follows. “Methodology” section presents the methodology. The results are presented in “Results” section. “Conclusions” section discusses the findings and concludes.

## Methodology

### Dependent and independent data

Our dependent variables are three forms of malnutrition among under-fives: stunting, underweight and wasting, selected from the Demographic and Health Survey (DHS^1^) conducted in 2016 [32] and 2009–2010 [55]. The selection of independent variables as potentially associated determinants was based on their correlation in the existing literature. The independent data that are used to explain the incidence of malnutrition and varying MDG-F JP impacts were based on the same DHS household surveys and on a set of geographical data. DHS surveys provided information on household characteristics, socio-economic conditions and personalized data of women and men of reproductive age and children under the age of five. Furthermore, DHS data include a geo-reference that marks latitude and longitude of households to geographical data sets of administrative units, agronomic characteristics and the prevailing natural resource base. The Mean Vegetation Health Index (MVHI) was used to assess the severity of drought during growing seasons, for each district separately [26]. Additional biophysical information on altitude, crop cover, population density, slope and soils of the household’s place of residence were retrieved from Buchhorn et al. [14]. By combining DHS data with the biophysical environment, we create a comprehensive analytical profile for our food security analysis.

### The geographical link

We established the geographical link between DHS data points and attribute maps in three steps. First attribute maps were rasterized to a 30 arcsecond resolution (app. 1 × 1 km) and resampled to the same geo-reference. A straightforward crossing between attribute and rasterized DHS point data retains only one single DHS observation per grid, leaving many DHS observations unattended. Hence, our second step applies a dedicated SAS program that exports gridded attribute maps into three vectors: latitude, longitude and attribute. In a third step we established the geographic link by seeking for each DHS point the nearest neighbor grid cell of the attribute maps.^2^ Removing observations with missing data creates a harmonized full matrix structure with attribute information from the DHS and geographic data. The format allows to conduct uni-, bi- and multi-variate analysis to explain children’s undernutrition in its geographical dependence on household characteristics, administrative entities, and biophysical variables. Furthermore, grouping data by administrative entities allows to conduct a quasi-experiment that assesses the MDG-F JP’s impact among different districts.

### Statistical analysis

The stunting, underweight and wasting among under-fives were represented in the DHS dataset by Z-scores lower than − 2.0, indicating the number of standard deviations (SD) below the mean of the WHO international reference standards [36, 66, 74]. Data processing and analysis were conducted with SAS 9.4 software.

First, univariate analyses were conducted to explore the distribution of undernutrition and other covariates among under-fives. The bivariate relationships between stunting, underweight and wasting and various covariates (as listed in Additional file 1) were tested with independent samples t tests for continuous variables and with MANOVA for categorical variables, using data from 2009 to 2010. For continuous determinants, scatterplots were generated beforehand, in order to assess distribution of these variables and opt for application of either the Pearson's correlation coefficient or the Spearman’s correlation coefficient [37]. Correlation was reported for variables having a correlation coefficient between (−) 0.10 and 1.0, in combination with statistical significance of *p* < 0.05 [17, 61]. To facilitate interpretation of the ranges of these continuous variables a categorical version of these variables was computed and included in the later performed multivariate regression analyses [41, 51]. Only if the bivariate analyses showed no statistically significant correlation with the (novel) categorical variable was the original continuous variable included. For bivariate regression analysis of dichotomous variables, the Folded F-test (α = 0.05) was applied to determine if the variances were indeed equal. If not (*p* < 0.05), the Satterthwaite output of the independent samples t test was examined instead of the Pooled output. For categorical determinants, the General Linear Model (GLM) procedure was used to perform multivariate analysis of variance (MANOVA), as the GLM procedure can also analyze unbalanced data. For each categorical variable, the *p*-value of the F-test was reported to assess whether the expected means of the variable’s subcategories differed significantly from each other. The R-square was reported to indicate how much variance was explained by each variable. By including an interaction term to each individual MANOVA of potential effect modifiers, relationships between independent variables were also examined.

Multiple linear regression models were generated using the General Linear Model Selection (GLMSELECT) procedure to find out which set of variables uniquely explained variance in stunting, underweight and wasting among the under-fives. In order to meet the multiple regression analysis linearity assumption, only biologically plausible and strong potential predictors (*p* < 0.05) based on bivariate analyses were included. A stepwise traditional backwards selection procedure was applied until only predictors with *p* < 0.05 were included, with a minimum number of 10–15 participants, while allowing split classification variables to leave the model independently. The resulting functional equation was tested for stability among different circumstances through sensitivity analyses [13, 15]. Model selection for HAZ-, WHZ-, and WAZ-scores was repeated on a total of 100 resampled subsets of the input data, each containing a randomly drawn 75% of the total sample, drawn without replacement [53]. Subsequently, average models were generated by averaging the parameter estimates of the selected models. Effects were dropped if they were not selected in at least 20% of the samples.

### Impact analysis

Based on our findings from the previous analytical exercise, we illustrate if and how changes have appeared over the years of the MDG-F JP interventions overall, stratified by the main determinants that were identified in the bi-variate and multivariate analyses. We analyzed trends, absolute scores and relative scores reported for 2009–2010 and 2016, as resulting from independent samples t tests and descriptive statistics. Subsequently, in order to analyze the MDG-F JP’s impact despite the unequal baseline scores, relative changes in HAZ, WAZ and WHZ means between 2009–2010 and 2016 among populations living in MDG-F JP districts were compared to those of populations not living in MDG-F JP districts. Within these independent populations, stratification was applied to create uniform groups that could be considered similar at the baseline, on the assumption of both resemblance of confounding structures and quasi-random exposure [16, 43]. There was no full random exposure, as the districts Aileu, Baucau, Manatuto and Oecusse were selected for the intervention due to their potential for optimizing the support and complementarities of existing programs and relationships. Baucau, Manatuto and Oecusse have previously been reported to be particularly susceptible to food insecurity and poor nutritional status [48]. More detailed information about the MDG-F JP can be found in Additional file 2.

## Results

### Sample characteristics

Anthropometric data, reported as height and weight, as well as HAZ, WAZ and WHZ-scores, were available for 7378 of the 9806 children under the age of five included in the DHS data file of 2009–2010 (missing data of 24.8%) and for 5081 of the 7221 children in the DHS data file of 2016 (missing data of 29.6%). A flow chart describing the study population selection process is provided as Additional file 3 and detailed descriptive statistics of the final study sample can be found in Additional file 4. Anthropometric characteristics are displayed in Table 1. As a result of natural variation, malnutrition occurs among 2.3% of under-fives in a well-fed population [69]. After subtraction of this percentage, the excess prevalence of stunting, underweight and wasting among under-fives in Timor-Leste is respectively 59%, 28% and 49% in 2009, and 51%, 41% and 29% in 2016. In our sample, 76% suffered from any form of malnutrition in 2009–2010, and 69% in 2016.

**Table 1:** Anthropometric characteristics of children in the sample

	2009–2010		2016	
	No	Pop. est	No	Pop est
Children in the sample	7378		5081	
HAZ, mean (SD)	6575	− 2.01 (1.95)	4603	− 1.70 (1.93)
WAZ, mean (SD)	6575	− 1.77 (1.25)	4817	− 1.64 (− 1.31)
WHZ, mean (SD)	6575	− 0.89 (1.61)	4608	− 0.96 (1.61)
Stunted	4504	61.05%	2680	52.75%
Underweight	2249	30.48%	2204	30.88%
Wasted	3745	50.76%	1569	43.38%
Any malnutrition (CIAF > = 1)	5600	75.90%	3480	68.49%

### Determinants of childhood undernutrition

For policy purposes we aimed to identify the main determinants that explain the status of malnutrition of under-fives. In the next sections we use bivariate and multivariate analyses for the detection of statistically significant explanatory variables.

### Bivariate analyses

The output of the Pearson’s and Spearman’s tests to explore correlation between HAZ-, WHZ-, and WAZ-scores and independent continuous variables is displayed in Additional file 5a, providing correlation coefficients and 95% confidence intervals. Weak statistically significant correlation (r > 0.1 and *p* < 0.05) was found between HAZ-scores and the MVHI of the first growing season (r = − 0.236; 95% CI – 0.259 to – 0.212); and birth weight (r = − 0.107; 95% CI 0.056 to 0.158). For WAZ-scores, birth weight (r = − 0.150; 95% CI 0.099 to 0.200); respondent's weight (r = − 0.148; 95% CI 0.124 to 0.172); the MVHI of season 1 (r = − 0.138; 95% CI − 0.162 to − 0.113); and respondent’s BMI (r = − 0.118; 95% CI 0.094 to 0.142) were weakly correlated. No significantly correlating continuous variables were found for wasting. Next, independent samples t tests showed whether Z-score population means varied significantly across groups composed on the basis of dichotomous variables. A brief overview of the results is shown in Table 2, with a focus on reporting categories with lower Z-scores than others, indicating increased severity of undernutrition. The complete output of population means, t-values, *p*-values and equality of variances is given in Additional file 5b.

**Table 2:** Bivariate analyses of dichotomous variables

Height-for-Age Z scores (HAZ)	Diff. means	*P*	Lower mean Z-score if:
Child received supplementary food	0.385	< 0.0001	Yes
Measured in hungry season	− 0.273	< 0.0001	No
Sex	− 0.210	< 0.0001	Males
Bicycle or motor vehicle	− 0.240	< 0.0001	No
Mother’s smoking status	0.286	0.0014	Yes
Respondent currently working	− 2.055	0.0242	No
Access to news media	− 0.110	0.0246	No
Owns livestock, herds or farm animals	0.238	0.0267	Yes
Sanitation facilities	− 0.107	0.0271	Non-improved
Source of drinking water	0.109	0.0309	Improved
Owns a bank account	0.217	0.0317	Yes
*Weight-for-age Z scores (WAZ)*			
Child received supplementary food	0.270	< 0.0001	Yes
Respondent currently working	0.199	< 0.0001	Yes
Type of place of residence	0.167	< 0.0001	Rural
Bicycle or motor vehicle	− 0.180	< 0.0001	No
Contraception use of mother	0.207	< 0.0001	Yes
Sex	− 0.087	0.0049	Males
Access to news media	− 0.071	0.023	No
Sanitation facilities	− 0.067	0.0285	Non-improved
*Weight-for-height Z scores (WHZ)*			
Respondent currently working	− 1.101	< 0.0001	Yes
Measured in hungry season	0.185	< 0.0001	Yes
Type of place of residence	0.157	0.0006	Rural
Contraception use of mother	0.151	0.0004	Yes
Child received supplementary food	0.081	0.042	Yes
Cow or bull owned	− 0.090	0.0357	No

Results from independent samples t tests using data from 2009–2010, indicating differences between population means (Diff. means) for HAZ-, WAZ-, and WHZ-scores for dichotomous variables, sorted by p-value (lowest) and t-value (highest)

Table 3 displays the categorical classification variables which were statistically significant associated with either HAZ-, WAZ- or WHZ-score population means, sorted by their statistical significance (see Additional file 5c). None of the classification variable estimates is estimable on their own. Lastly, by including an interaction term to each individual MANOVA of potential effect modifiers, relationships between the independent variables were examined, as displayed in Additional file 6. Child age was significantly related (*p* < 0.05) to 50% of other variables when predicting stunting, and to 75% of other variables when predicting wasting and underweight. Wealth was an important effect modifier only for underweight (75%).

**Table 3:** Bivariate analyses of categorical variables

Height-for-age Z scores (HAZ)	Pr > F	Lowest Z-score
District	< 0.0001	Bobonaro
Age of child	< 0.0001	3
MVHI cat	< 0.0001	Third quantile*
Month of birth	< 0.0001	February*
BMI class	< 0.0001	Underweight
Household size cat	< 0.0001	6–10*
Wealth	< 0.0001	Poorer
Agricultural land cat	0.0002	> = 3 ha*
Partner’s highest educational level	0.0006	No education
slope	0.0018	8–16%
soils	0.002848	Moderate
Mother’s highest educational level	0.0040	None
Partner’s occupation	0.0113	Agric-employee
Size of child at birth	0.0133	Smaller than average
Child’s anemia level	0.0330	Mild
*Weight-for-age Z scores (WAZ)*		
Age of child	< 0.0001	4
District	< 0.0001	Oecusse
Month of birth	< 0.0001	April*
BMI class	< 0.0001	Underweight
Mother’s occupation	< 0.0001	Skilled manual*
Wealth	< 0.0001	Poorest
Mother’s highest educational level	< 0.0001	None
Size of child at birth	< 0.0001	Smaller than average
Partner’s highest educational level	< 0.0001	No education
MVHI cat	< 0.0001	Third quantile*
soils	0.0002	Moderate
Household size cat	0.0004	6–10
Partner’s occupation	0.0030	Agric. Employee*
Altitude cat	0.0059	> 1200
Population density cat	0.0146	> 500
*Weight-for-height Z scores (WHZ)*		
District	< 0.0001	Aileu
MVHI cat	< 0.0001	Second quantile
Age of child	< 0.0001	4
Respondent’s occupation	< 0.0001	Skilled manual
Size of child at birth	< 0.0001	Average
BMI class	< 0.0001	Underweight
slope	0.0006	16–30%
Wealth	0.0016	Middle*
Agricultural land cat	0.0043	No land
Partner’s occupation	0.0055	Prof, Tech, Manag
Altitude cat	0.0055	800–1000*
Mother’s highest educational level	0.0139	Primary*
Partner’s highest educational level	0.0283	Higher*
Month of birth	0.0482	December*

Based on data from 2009–2010. Categorical variables are sorted by p-value (lowest) and R-square (highest)

*Statistically significant association with the overall variable, but not with the specific class

### Multiple linear regression models

Explanatory modelling entailed a backwards selection procedure and multivariate regression analysis for each dependent variable, whilst pre-selecting variables that were reported to be related to HAZ-, WHZ-, and WAZ-scores in bivariate models. The relationships between independent variables and HAZ-, WAZ-, and WHZ-scores from multiple linear regression analysis are shown in Table 4, and entirely in Additional file 7. The adjusted R-Square of the regression model for HAZ-scores was 0.1091, for WAZ-scores was 0.1859 and for WHZ-scores was 0.1071. During the repeated model selection procedure, each covariate in the main models appeared to have been selected in at least 20% of the samples (as shown in Additional file 8) and main model parameter estimates did not deviate significantly from the averaged samples’ estimates. Covariates in the main models were therefore considered robust explanatory variables.

**Table 4:** Explanatory regression model of HAZ-, WAZ- and WHZ-scores

Parameter		HAZ^1^-scores		WAZ^2^-scores		WHZ^3^-scores	
		Coeff	*p*-value	Coeff	*p*-value	Coeff	*p*-value
Intercept		− 1.29	< 0.0001	− 1.31	< 0.0001	− 1.54	< 0.0001
Measured in hungry season	No	*Reference category*					
	Yes	0.28	0.0275			− 0.13	0.0376
Sex	Male	*Reference category*					
	Female	0.35	0.0032	0.11	< 0.0001		
District	Aileu	*Reference category*					
	Ainaro	− 0.55	0.0026	− 0.20	< 0.0001	1.45	< 0.0001
	Baucau					0.95	< 0.0001
	Bobonaro			− 0.24	< 0.0001	1.26	< 0.0001
	Cova Lima	− 1.02	0.0007	− 0.18	0.006	0.85	< 0.0001
	Dili	− 0.97	0.0108			1.34	< 0.0001
	Ermera	− 1.90	< .0001	− 0.50	< 0.0001	0.93	< 0.0001
	Lautem					1.01	< 0.0001
	Liquica					0.98	< 0.0001
	Manatuto	0.87	< .0001			0.73	< 0.0001
	Manufahi					1.18	< 0.0001
	Oecusse			− 0.54	< 0.0001	0.58	< 0.0001
	Viqueque					0.91	< 0.0001
Age of child	Zero	*Reference category*					
	One	− 1.03	< .0001	− 0.82	< 0.0001	− 0.64	< 0.0001
	Two	− 0.77	0.0008	− 1.04	< 0.0001	− 0.74	< 0.0001
	Three	− 1.02	< .0001	− 1.14	< 0.0001	− 0.62	< 0.0001
	Four	− 0.83	0.0003	− 1.22	< 0.0001	− 0.90	< 0.0001
Month of birth	Jan	*Reference category*					
	March	− 0.45	0.0352				
	June			0.12	0.019		
	July			0.24	< 0.0001		
	Aug			0.20	0.000		
	Sept			0.42	< 0.0001		
	Oct			0.48	< 0.0001	0.25	0.0156
	Nov	0.57	0.0129	0.45	< 0.0001		
	Dec			0.33	< 0.0001		
Slope	8–16%	*Reference category*					
	16–30%	0.55	0.0008				
Soil suitability	Marginal	*Reference category*					
	Moderate	− 0.90	< 0.0001				
Partner’s occ	Prof. Tech. …	*Reference category*					
	Agric-self employed	− 0.32	0.0139			0.20	0.0056
	Services					0.20	0.0408
Suppl. food	No	*Reference category*					
	Yes			− 0.16	< 0.0001		
Sanitation facilities	Non-improved	*Reference category*					
	Improved			− 0.08	0.017		
BMI class	Underweight	*Reference category*					
	Normal			0.33	< 0.0001	0.29	0.0001
	Overweight			0.47	< 0.0001	0.45	0.0011
Wealth	Poorest	*Reference category*					
	Poorer			0.08	0.024		
	Richer			0.14	0.001		
	Richest			0.23	< 0.0001		
Size of child at birth	Average			0.12	0.004		
	Larger			0.22	< 0.0001	0.21	0.0038
Altitude	= < 800 m	*Reference category*					
	800–1000 m					0.31	0.038
Mother’s occ	Not working	*Reference category*					
	Prof. Tech. …			0.24	0.027		
Household size	0–5	*Reference category*					
	6–10			− 0.07	0.017		
Pop. dens	= < 50	*Reference category*					
	> 500			− 0.24	< 0.0001		
Contraception use	Non-user	*Reference category*					
	User					− 0.13	0.0487

Based on data from 2009–2010

^a^HAZ, height-for-age Z-score, indicator of stunting; ^b^WAZ, weight-for-age Z-score, indicator of underweight; ^c^WHZ, weight-for-height Z-score, indicator of wasting

### MDGF-JP impact

At baseline, in 2009–2010, average HAZ-scores were higher in MDG-F JP districts (diff. between means: − 0.778; 95% CI − 0.885 to − 0.671), indicating less severe stunting, whereas average WHZ-scores were lower in MDG-F JP districts (diff. between means 0.661; 95% CI 0.579 to 0.743), indicating more severe wasting. No significant difference with non-MDG-F JP districts was found for WAZ scores, indicating underweight. At end line, after the MDG-F JP, in 2016, no statistically significant difference in mean HAZ-, WHZ-, and WAZ-scores between MDGF-JP and non-MDG-F JP districts appeared. As we know from our absolute descriptive results, overall, in MDGF-JP and non-MDG-F JP districts together, average severity of stunting and underweight decreased between 2009 and 2016, but wasting severity increased.

With regard to change of Z-scores over the years in MDG-F JP and non-MDG-F JP districts, outcomes are displayed in Tables 5 and 6, and more deliberately in Additional file 9. With regard to stunting, in general, living in an MDG- F JP district led to an unfortunate average decrease of HAZ-scores (Abs.: − 21; rel. − 14%), whereas not living in MDG-F JP districts led to a fortunate increase of HAZ-scores (Abs. + 57; rel. + 25%). Within groups classified by each independent variable, positive impact of the MDG-F JP on Z-scores was only found for children whose mother had overweight (No JP: + 35.729 (17%) and JP: + 39.830 (20%)), with an absolute difference in Z-score increase of 4.101. Positive impact was also reported for children whose father was an agricultural employee (No JP: + 99.847 (36%) and JP: + 115.412 (59%), with an absolute difference), or a skilled manual (No JP: + 27.272 (13%) and JP: + 59.055 (25%), with an absolute difference of 31.78%), but the populations in the comparison were too small to reliably report differences (< 50).

**Table 5:** Impact of the MDG-F JP-overall

	No MDG-F JP	MDG-F JP	Impact
	2009 → 2016	2009 → 2016	
Height-for-Age Z-score (stunting)	+ 0.57 (25%)	− 0.20 (− 14%)	− 0.77
Weight-for-Age Z-score (underweight)	+ 0.14 (8%)	+ 0.12 (7%)	− 0.02
Weight-for-Height Z-score (wasting)	− 0.30 (− 45%)	+ 0.37 (28%)	+ 0.66

Overview of the development of Z-scores in districts where no MDG-F JP was implemented versus where an MDG-F JP was implemented

**Table 6:** Impact of the MDG-F JP-specified

		Z-score increase				
		No JP		JP		
		Abs. Diff	%	Abs. Diff	%	Abs. diff
*HAZ-scores (indicating stunting)*						
*Overall*		57	25	− 20	− 14	− 77
BMI class	Overweight	36	17	40	20	4.1
*Partner’s occ*	*Agric-employee*	*100*	*36*	*115*	*59*	*16*
	*Skilled manual*	*27*	*13*	*59*	*25*	*32*
*WAZ-scores (indicating underweight)*						
Overall		14	8	12	7	− 2.2
Owns livestock	No	0.36	0	38	23	38
Males–females	Females	7.3	5	37	20	30
Age of child	1	9.7	6	18	10	8.0
	2	16	9	17	9	0.49
	3	9.0	4	26	12	17
BMI class	Underweight	19	9	37	17	18
	Overweight	7.6	5	37	21	30
Wealth	Richer	1.4	1	13	7	11
	Richest	20	14	39	22	19
Agricultural land	> = 3 ha	9.6	6	35	19	25
Mother's highest edu	Primary	2.3	1	21	11	19
	Incomplete sec	2.5	1	13	8	11
	Complete sec	0.12	0	13	8	12
*Partner’s occ*	*Skilled manual*	*6.2*	*4*	*36*	*17*	*30*
	*Unskilled manual*	*5.3*	*3*	*11*	*7*	*5.5*
Size of child at birth	Smaller	− 17	− 9	27	13	45
*WHZ-scores (indicating wasting)*						
Overall		− 30	− 45	37	28	66
Owns livestock	No	− 48	− 72	71	49	119
	Yes	− 27	− 40	26	21	53
Males–females	Males	− 28	− 39	27	20	55
	Females	− 31	− 52	48	36	79
Type of place of residence	Urban	− 28	− 43	33	27	61
	Rural	− 31	− 46	36	27	67
Current age of child	0	− 47	− 349	29	34	76
	1	− 33	− 47	31	26	63
	2	− 23	− 33	40	27	63
	3	− 34	− 48	51	36	86
	4	− 15	− 16	23	15	38
BMI class	Underweight	− 43	− 47	37	24	80
	Normal weight	− 31	− 50	34	27	66
	Overweight	− 21	− 50	27	29	48
Wealth	Poorest	− 28	− 44	17	13	45
	Poorer	− 45	− 78	34	28	79
	Middle	− 16	− 22	41	29	58
	Richer	− 40	− 63	52	36	93
	Richest	− 16	− 23	37	35	53
Agricultural land	*No land*	− *17*	− *20*	*72*	*43*	*89*
	> = 3 ha	− 42	− 73	39	32	81
Mother’s highest edu	None	− 34	− 53	30	23	64
	Incomplete primary	− 44	− 70	33	23	77
	Primary	− 24	− 35	20	14	44
	Incomplete sec	− 29	− 44	42	31	72
	Complete sec	− 32	− 49	16	17	48
	Higher	− 0.5	− 1	73	46	73
	Prof., Tech., Manag	− 17	− 22	36	27	53
	Agric-self employed	− 48	− 73	40	30	88
	*Skilled manual*	− *14*	− *16*	− *1*	− *1*	*12*
	*Unskilled manual*	− *45*	− *74*	*24*	*25*	*69*
Size of child at birth	Smaller	− 45	− 58	47	34	92
	Average	− 21	− 29	52	35	73
	Larger	− 44	− 110	17	17	60

Development of Z-scores in districts where no MDG-F JP was implemented versus where an MDG-F JP was implemented, additionally reported for sub-groups separately, classified by significantly related covariates. Sample sizes smaller than 50 children are indicated in italics. Z-score changes are presented with two implied decimal places

For underweight, living in MDG-F JP districts had a weaker positive impact on the whole population’s WAZ-scores than not living in MDG-F JP districts. Only within some specific groups a positive impact of MDG-F JP appeared, namely in groups of children whose household did not own livestock, herds or farm animals; girls; children living in urban regions; children who were 1, 2 or 3 years old; children of mothers who were underweight or overweight; children in the two richest wealth quintiles; children whose parents own more than three hectares of land; children whose mother had primary, incomplete secondary, or complete secondary education; and lastly children who were smaller than average at birth. The groups of children whose father was a skilled or unskilled manual worker also seemed to have profited from living in an MDG-F JP district. However, groups were too small to report such differences. A positive impact of MDG-F JP for wasting was found for the overall population, as well as in every uniform sub-population. For groups with missing measurements or insufficient population size (agricultural land and partner’s occupation), no positive impact could be reported.

## Discussion

### Contributions of the MDG-F Joint Program

For the evaluation of the impact of the MDG-F Joint Program we compared the nutritional status of the study population before and after the program. In districts in which the MDGF-JP had been implemented, the baseline situation was better in terms of stunting than in non-JP districts (Diff. HAZ-score means: + 0.8). After the program ended, HAZ-score means were similar to those in non-MDG-F JP districts. The fact that HAZ-scores increased in non-JP districts (+ 25%) but decreased in JP districts (− 14%) implies a negative and inverse effect of the MDG-F joint program, potentially caused by persistent factors causing stunting, and/or time- and place-specific adverse environmental factors [9].

For underweight, the situation at baseline and end line was approximately similar in JP and non-JP districts, as no statistically significant differences were found in 2009 and in 2016. For the sample as a whole, the mean WAZ-score increased between 2009 and 2016. No evidence of either a positive or negative impact of the MDG-F JP on reducing population-wide underweight was found. Non-MDG-F JP districts had an HAZ-increase of 0.14 (+ 8%) and MDG-F JP districts had an increase of 0.12 (+ 7%) over the years. However, when moving from a population-wide scope to a comparison of similar groups across intervention and control districts, it is striking to notice an increase of WAZ-scores in specific groups within the MDG-F JP districts that is greater than among similar groups in non-MDG-F JP districts. Specifically, increased WAZ-scores were found for children whose mothers had primary, incomplete secondary, or complete secondary education; children living in urban areas; and for children in the two richest wealth quintiles. The fact that only a limited group seems to have profited from the program might very well indicate that farmers’ groups and mother support groups and the new agriculture and nutrition practices and methods that are taught and provided by the MDG-F program are initially adopted by the better educated and wealthier groups. Reaching non-literate and remote farmers and mothers appears to be a major challenge, which has also been acknowledged in an earlier MDG-JP evaluation report [44]. There is a need for further examination regarding the high variation in the impact of the MDG-F JP’s on different populations’ WAZ-scores.

In terms of wasting, the situation was much more problematic in JP districts than in non-JP districts (WHZ-scores in JP districts: − 1.32; non-JP districts: − 0.7; diff: 0.7) at baseline, but similar at end line (both − 0.96). In the overall population, WHZ-scores fell significantly, representing increased severity of wasting. Despite the average decrease of WHZ-scores, the absolute prevalence of children classified as wasting has slightly increased in the overall population. Regarding the different trends, a negative WHZ-score trend was observed in non-MDG-F JP districts (abs. − 0.3; rel. − 45%) while a positive trend was observed in MDG-F JP districts (abs. + 0.37; rel. + 28%).

The contrast between the obvious lack of evidence for impact of the MDG-F JP on stunting and the observed positive trends regarding wasting-rates has many possible interpretations. First, stunting is considered a chronic form of undernutrition and is only associated with a child’s height, which cannot easily be addressed within a period of a few years, in contrast to wasting. Childhood stunting remains a strongly persistent problem in Timor-Leste compared to global trends, but the underlying causes are not yet understood [19]. It is suggested that maternal problems in Timor-Leste play a substantial role, confirming the importance of MDG-F JP's focus on mothers, but also highlighting the importance of further examination of underlying factors [19]. Although wasting and stunting are closely related, each represents a unique health threat. Wasting is associated with a greater risk of mortality, but there is also the possibility of treatment. Episodes of wasting seem to increase the risk of stunting in particular contexts, but evidence is mixed [40]. Therefore, in a sense, the decline in the incidence of wasting offers perspectives, but a broader range of socio-economic and maternal determinants plays a role in its occurrence and prevention.

Lastly, the lack of evidence for (un-)successful impact of the MDG-F JP on Z-scores can be largely explained by baseline differences and district-specific (unobserved) confounders. For instance, wealth appeared to be an important effect modifier when assessing underweight, and was also correlated with district of residence. The rationale behind the districts selected for specific program components remains largely unspecified. The uneven distribution of Timor Vita across MDG-F JP districts, for example, could have played a role [46]. Such unobserved contextual and program confounding factors challenge the demarcation of the MDG-F JP initiatives and their impact.

### Determinants of child nutritional status

In order to develop relevant policy recommendations, we explored the context-specific determinants of food security. Regarding child age, the data showed that stunting and underweight were more severe as the child became older, corroborating previous studies [11, 31]. Next, in line with the literature, our findings suggest a major negative impact of various maternal health factors on children’s nutritional status, such as smoking [8, 62] and underweight [35]. Our findings show that both underweight and wasting appeared to be significantly more severe in children whose mothers use contraception. Previous studies have yielded mixed results on this subject [20, 30, 59]. Parents’ lack of education, occupation and wealth proved to be important determinants of childhood undernutrition both in current and previous studies [29, 31, 64]. Lastly, the literature commonly cites water quality and sanitation as important determinants of how food is prepared and children’s nutritional status [24, 29, 58]. It was therefore alarming to find that that fewer people had access to an improved source of drinking water in 2016 than in 2009–2010 (59% vs 26%), and access to improved sanitation facilities decreased from 48 to 33%. Yet, this study found mixed associations between the use of water and sanitation facilities and undernutrition, which accords with earlier studies by Fagbamigbe et al. [29] where complex structure of (confounding) factors blurred possible causal relationships [57].

### Policy recommendations

The outcomes of current study reinforce the complex multi-factorial nature of undernutrition among under-fives in SIDS, and the pressing need for integrated and cross-sectoral programs. Integration is important, in order to effectively reach specific populations, namely mothers and children. Their situation is strongly interconnected with broader population health issues and far-reaching developmental consequences of undernutrition in early life; and an intergenerational cycle of poor health can potentially be avoided [4, 10, 39, 75]. According to this study, young women who are underweight and are particularly likely to have underweight children should receive nutritional support, for example during pregnancy [35]. The integrated focus on mothers and children aligns with the priorities set by local political authorities [19, 35]. Households’ dependence on agricultural assets and activities also seems to increase their vulnerability to undernutrition. Therefore, interventions should be adapted to occupational, biophysical and socio-economic context and prioritized based on prevalence of malnourished children.

Reduction of undernutrition cannot be solved merely by health and nutrition interventions [9], but also requires better linking with the agricultural sector [75]. The MDG-F JP was mainly focused on nutrition security, and so took an integrated approach only to a limited extent [44]. The agricultural challenge, however, is not only to increase food production, but also to sustainably produce better-quality and health-enhancing foods [38]. Therefore, in the optimal case, programs should combine agricultural components with complementary non-agricultural components, like education, on maternal health-seeking, caring and nutritional practices [2]. Better education is needed in the broad sense, as well as being focused on agriculture. Both the local experts interviewed for this study and previous studies indicate that there is a lack of alignment of research, government budgeting, farmers’ practices, local wisdom and extension workers’ ideas in Timor-Leste [19]. Therefore, participatory research and education on (the adoption of) high-yield and highly nutritious crop varieties need specific attention and should combine external resources with farmers’ existing livelihood systems and environmental context, and address improving farmers’ conservation and seed-preservation techniques, as well as flexible recommendations and long-term commitment of aid agencies [24, 56, 67, 76].

Programs encouraging participatory selection of seed varieties have proved effective in giving farmers ownership [42]. Examples of improved agricultural practices are intercropping, replacing traditional slash and burn (swidden) agriculture and seasonal bush burning [47]. These developments would contribute to achieving the goals of the Timor-Leste’s Strategic Development Plan, stating that by 2030 every citizen has the opportunity to acquire new skills based on 21st-century technologies, such as high-yield agriculture [33]. Crop selection was reported as part of the MDG-F JP, albeit to a limited and barely described extent [71]. For the MDG-F JP, constraints for sustainable outcomes were also found in institutional cooperation [44]. As part of the MDG-F JP, public–private partnerships (PPPs) were set up for production of fortified food supplements at the local level [71]. However, those who attended were often unfamiliar with the prescribed methods and formats, which constrained efficient cooperation and action [71]. The next linkage is therefore to increase cooperation with, and capacity of, local community-level organizations, to be able to explore and access new technologies, as well as to set up community-based seed businesses. This contributes to the sustainability of efforts often made by aid-supported programs [12, 23, 38].

The impact of being either born or measured in Timor-Leste’s hungry season on nutritional status was striking. Despite the fact that globally, acute hunger and undernutrition are more prevalent in annual hunger seasons than in conflicts and natural disasters, it receives far less public attention and underlines the importance of intelligent and context-specific selection of crop varieties [7, 72]. There is also a need for food price regulations and improved storage techniques to prevent households from falling into seasonal hunger. In addition, this would reduce the country’s increasing dependence on imported food, seeds and fertilizers. The promotion and protection of regional markets and smallholders’ businesses is needed to prevent great transport distances and unfair prices not covering full production costs [34]. To that end, despite the lack of details, the MDG-F JP was reported to have improved access to markets, which in turn has led to a more varied diet and greater food sovereignty [34, 71]. After all, in Timor-Leste as on other SIDS, developments should entail a self-defined food and agricultural system, putting those who produce, distribute and consume the food at the core of the system and policies, while also being healthy, culturally appropriate, ecologically sound and sustainable [34]. In addition, this includes promoting the preservation of local seeds, fish, and livestock varieties, dietary diversity through traditional foods, and the support of local innovations and the value-adding development of local agri-processing and marketing [34].

## Conclusions

We found that undernutrition is seasonal and locational, and that in addition, sex, age, size at birth, mother’s weight and wealth are major contributing factors to undernutrition in this group of under-five children at the household and individual level in Timor-Leste. There has been a drop in the severity of wasting over the years in districts in which the MDG-F JP was implemented, whereas stunting severity has unfortunately increased in these districts. Both run counter to trends seen in the overall population of under-fives in Timor-Leste. In theory, the MDG-F JP (partially) included many components that are necessary for a successful program, such as integration, cooperation, prioritization of children and young mothers, linking of nutrition and agriculture, and support of local community-level organizations and innovations. At the same time, one of the (potential) flaws in the MDG-F JP and other food-security programs, is the lack of policy and cooperation embracing different sectors, as well as the application of participatory research and education. Climate change, population growth and persistent failure to generate sufficient food and revenues on at the macro and micro level also reinforce the need to strengthen the focus on the selection and adoption of high-yield and highly nutritious crop varieties, on improved agricultural practices, and on facilitation of agri-processing industries and markets.

## Strengths and limitations

The observational nature of this study did not allow for unambiguous determination of causality and increased the probability of residual confounding effects. Moreover, the use of anthropometric characteristics as indicators for food insecurity aligned with the literature, but might not necessarily have been a flawless classification strategy, as it does not completely recognize the complex interaction between food security and health status [28, 68]. As a rule, an increase of mean Z-scores was indicated as positive in this study, without taking account of where on the growth chart individual increases occurred. Likewise, validity issues occurred in other variables, for example in the asset-based index of household wealth and the minimum dietary diversity, resulting in weak or non-detectable associations. Due to a lack of integrated data sources, biophysical variables were aggregates from districts (MVHI) or clusters (altitude, slope, population density, soil suitability), of which the timing data collection was not exactly similar to the survey data collection. Similarly, the timing of DHS-data collection did not exactly conform to the baseline and end line of the MDG-F JP. Determination of the MDG-F JP’s actual impact remained a challenge, despite the use of representative DHS surveys, a comprehensive set of spatially tailored covariates, and the quasi-experimental design partially decreasing residual confounding and increasing precision [18].

A strength of this study is its careful selection of variables based on bivariate analyses of scientifically plausibly related variables, in order to prevent model-selection issues like data dredging and overfitting [3, 51]. Also, the thorough sensitivity analyses supported the primary findings and thus confirm reliability. Our multiple linear regression models had low adjusted R-square values of between 0.11 and 0.19. This is, however, not unusual in social research. Moreover, the model was used to identify explanatory variables, but not to provide a full predictive model of undernutrition among under-fives [1, 49]. Due to the use of context-specific variables and the specific program assessed in the current study, generalizability of outcomes to other countries and programs is limited, although the methodology may inspire future program evaluations. Moreover, the spatially embedded findings ideally meet the need for tailored evidence on health and socio-demographic indicators, applicable to local, national and international purposes. Furthermore, many challenges that generally face SIDS are reflected in this sketch of the situation in Timor-Leste. SIDS are on the frontlines of climate change, and many climate-related challenges described in this paper merit increasing attention [60].

## Future research

The current study has given an exploratory outline of the way in which spatial, temporal and interpersonal variation plays a role in both the risk of undernutrition and the impact of interventions. There is a need for more in-depth geo-spatial, socio-economic and institutional research to facilitate a precise alignment of future policies and programs for specific contexts. For example, an improved empirical base could explain how agricultural assets and production of individual households affect undernutrition in Timor-Leste. Especially in SIDS, there is a need for a rapid increase of nutrient-rich food production to tackle the problem of persistent food-insufficiency, in addition to population growth and climate change. Therefore, there is a need for participatory agricultural enhancement studies to facilitate self-supply based on sustainable food production systems. Next, studies in which households actively participate through surveys and focus group discussions (FGDs) would offer insights into sustainable and effective livelihood strategies on child-feeding practices. Furthermore, to increase food self-sufficiency in Timor-Leste as a nation, as well as for individual farmers, it would be useful to explore farmers’ market mechanisms and (potential) revenue models, as the local food markets seem unable to supply food that is both affordable and profitable. Technology and agri-processing industries have the potential to improve sustainable production and conservation, but should again be affordable, profitable and adoptable, and should therefore be properly considered and facilitated through participatory research and policymaking. Given that stunting appears to be a persistent problem over the years, especially in Timor-Leste and despite the MDG-F JP and decline in wasting, there is a clear need for further exploration of interventions that might curb the intergenerational cycle of growth failure. Lastly, the way in which seasonality and maternal (pre-) pregnancy health status determine the perfect timing of interventions should be further explored.

### Supplementary Information

**Additional file 1**. Overview of variables.

**Additional file 2**. Background information on the MDG-F JP.

**Additional file 3**. Flow chart of the study population.

**Additional file 4**. Characteristics of children in the sample.

**Additional file 5**. Bivariate analyses.

**Additional file 6**. Interaction terms.

**Additional file 7**. Multiple linear regression models.

**Additional file 8**. Model selection.

**Additional file 9**. MDG-F JP impact.

## Data Availability

The datasets analyzed during the current study are available in the DHS repository, https://www.dhsprogram.com/data/dataset/Timor-Leste_Standard-DHS_2009.cfm and https://www.dhsprogram.com/data/dataset/Timor-Leste_Standard-DHS_2016.cfm.

## Abbreviations

DHS: Demographic and Health Survey
HAZ: Height-for-age Z-scores
JP: Joint Program
MDG-F: Millennium Development Goals Achievement Fund
MDG-F JP: Millennium Development Goals Achievement Fund Joint Program
MDGs: Millennium Development Goals
MVHI: Mean Vegetation Health Index
SIDS: Small Island Developing State
WAZ: Weight-for-age Z-scores
WHZ: Weight-for-height Z-scores

## Footnotes

1 Sampling of DHS participants was based on the 2004 Timor-Leste Population and Housing Census, which has yielded independent samples from rural and urban strata in all 13 districts [55].

2 For each DHS data point the distance to the attribute grid was calculated. A descending sort, next, identified the nearest neighbor of the DHS points in the attribute map.
