# Supplementary material for: Analyzing the impact of an MDG-Fund program on childhood malnutrition in Timor-Leste
Source: J Health Popul Nutr. 2024 Apr 4;43:46. doi: 10.1186/s41043-024-00539-x (PMC10993443; doi:10.1186/s41043-024-00539-x)
Supplement: Supplementary file 1 — Additional file 1. Overview of variables. [file 41043_2024_539_MOESM1_ESM.pdf]

Article: *Analyzing the impact of an MDG-Fund program on childhood malnutrition in Timor-Leste;*
Journal: *Environment, Development and Sustainability;*
Authors: *H.D. van der Spek, MSc. (lindavdspek@live.nl) and Dr. B.G.J.S. Sonneveld (b.g.j.s.sonneveld@vu.nl).*

Online Resource 1: Overview of variables

**Children's characteristics**			
**Dependent variables**			
HW70	Ht/A SD's (according to WHO)	AV8[1]	Underweight
HW71	Wt/A SD's (according to WHO)	AV9[1]	Wasting
HW72	Wt/Ht SD's (according to WHO)	AV11[1]	CIAF (Composite Index of Anthropometric Failure)
AV7[1]	Stunting		
**Continuous variables**			
M19	Birth weight	M35	Times breastfed during night
B16	Child's line number in household	M36	Times breastfed during day
HW56	Hemoglobin level adj. by altitude	AV17[1]	Times breastfed in last 24hrs
**Dichotomous variables**			
AV15[1]	MDD (Minimum Dietary diversity)	AV18[1]	Child received supplementary food
AV16[1]	Hungry season (Month of measurement)	B4	Child's sex
**Categorical variables**			
B1	Month of birth	HW57	Child's anaemia level
B8	Age of child	M18a[1]	Size of child at birth
**Indirectly employed variables[3]**			
HW18	Date measured (month)	V414J	Gave child any dark green leafy vegetables
M18	Size of child at birth	V414K	Gave child mangoes, papayas, other vitamin A fruits
S517A	Child ever received suppl. food	V414L	Gave child any other fruits
V414E	Gave child bread, noodles, other made from grains	V414M	Gave child liver, heart, other organs
V414F	Gave child potatoes, cassave, or other tubers	V414N	Gave child fish or shellfish
V414G	Gave child eggs	V414O	Gave child food made from beans, peas, lentils, nuts
V414H	Gave child meat	V414P	Gave child milk products
V414I	Gave child pumpkin, carrots, squash (yellow or orange inside)		
**Residential characteristics**			
**Continuous variables**			
AV13A[1]	MVHI1 (S1)	HV246F	Chickens own
AV13B[1]	MVHI2 (S2)	V136	Number of household members
HV246B	Cows, bulls own	Altit[2]	Altitude
HV246C	Horses, donkeys, mules own	Cropc[2]	Crop cover: Share of percentage cultivated land, multiply by 10.
HV246D	Goats own	Popde[2]	Population density
HV246E	Sheep own		
**Dichotomous variables**			

AV2[1]	Access to news media	AV23b[1]	Horse, donkey or mule owned
AV13[1,2]	MDGF JP	AV23c[1]	Goat or sheep owned
AV19[1]	Source of drinking water	AV23d[1]	Chicken owned
AV20[1]	Sanitation facilities	HV246	Owns livestock, herds or farm animals
AV21a[1]	Motor vehicle or bicycle owned	HV247	Owns a bank account
AV23a[1]	Buffalo, cow or bull owned	V140	De jure type of place of residence (urban/rural)
**Categorical variables**			
AV24[1]	Household size cat	V139	De jure region of residence (District)
AV25[1]	Agricultural land cat	AV33[1,2]	Cropcover cat
AV26[1]	Population density cat	AV35[1,2]	Soil suitability
AV27[1]	Altitude cat	AV36[1,2]	Slope
AV28[1]	MVHI cat		
**Indirectly employed variables[3]**			
V113	Source of drinking water	V125	Has car/truck
V116	Type of toilet facility	Slope[2]	Slope
V123	Has bicycle	Soils[2]	Soil suitability
V124	Has motorcycle/scooter	HV245	Hectares for agricultural land
**Parents' characteristics**			
**Continuous variables**			
V437	Respondent's weight	V445	Body mass index for respondent
V438	Respondent's height		
**Dichotomous variables**			
AV4[1]	Contraception use of mother	V714	Respondent currently working
AV6[1]	Mother's smoking status	V151	Female-headed household
**Categorical variables**			
AV22a[1]	Mother's BMI class	V701	Partner's highest educational level
V149	Mother's highest educational level	V705	Partner's occupation
V190	Wealth	V717	Respondent's occupation
V457	Mother's anemia level		
**Indirectly employed variables[3]**			
V191	Wealth index factor score	V463C	Chewing tobacco
V313	Current use by method type	V463D	Uses snuff
V463A	Smokes cigarettes	V463E	Smokes rolled tobacco
V463B	Smokes pipe		
**Identification variables[3]**			
V135	Usual resident or visitor	V000	Country code and phase
B9	Child lives with whom	V001	Cluster number
B5	Child is alive	V002	Household number
BORD	Birth order number	V003	Respondent's line number
CASEID	Case Identification	V004	Ultimate area unit
HHID	Case Identification	V005	Sample weight
HIDX	Index to birth history	V021	Primary sampling unit
HV004	Ultimate area unit	V022	Sample stratum number
HV005	Sample weight	V023	Sample domain

2009-2010 variable information retrieved from Rutstein & Rojas (2006); 2016 variable information retrieved from Croft et al. (2018). [1]These variables were additionally computed. [2] These variables were not retrieved from the DHS-datasets, but from Buchhorn et al. (2020) and FAO (2020). [3]These variables were not used for statistical analyses.
