# Supplementary material for: Analyzing the impact of an MDG-Fund program on childhood malnutrition in Timor-Leste
Source: J Health Popul Nutr. 2024 Apr 4;43:46. doi: 10.1186/s41043-024-00539-x (PMC10993443; doi:10.1186/s41043-024-00539-x)
Supplement: Supplementary file 2 — Additional file 2. Background information on the MDG-F JP. [file 41043_2024_539_MOESM2_ESM.pdf]

Article: *Analyzing the impact of an MDG-Fund program on childhood malnutrition in Timor-Leste;*
Journal: *Environment, Development and Sustainability;*
Authors: *H.D. van der Spek, MSc. (lindavdspek@live.nl) and Dr. B.G.J.S. Sonneveld (b.g.j.s.sonneveld@vu.nl).*

Online Resource 2: Background information on the MDG-F JP

This study focused on the impact of the MDG-F Joint Program on Promoting Sustainable Food and Nutrition Security in Timor-Leste. The Millennium Development Goals-Fund financed a total amount of 130 collective action initiatives on poverty and reaching the MDGs in 50 separate countries since 2007. The MDG-F was created when the Spanish government signed an agreement with the UN, in particular with the United Nations Development Program (UNDP). Four different UN organizations were involved, of which UNICEF became the lead agency. In Timor-Leste, the first funds for this particular 36-months-program were provided in November 2009, which was later extended with 18 weeks, ending on the 31st of March 2013 (Lenci, 2012). A factual overview of the program is given in table 1.

TABLE 1: FACTUAL OVERVIEW OF THE MDG-F JP[1]

**Budget**	3,500,000 USD<br>Provided by the MDG-F in three disbursements:<br>- 1,741,960 USD (17 December 2009)<br>- 1,143,432 USD (13 April 2011)<br>- 614,608 USD (10 January 2012)<br>Allocated to participating UN agencies (Lenci, 2012):<br>- UNICEF: 2,277,856 USD<br>- WFP: 720,645 USD<br>- FAO: 447,999 USD<br>- WHO: 53,500 USD<br>An additional 530,000 USD was provided by the Government of Timor-Leste.
**Participating UN agencies**	- UNICEF<br>- WHO<br>- FAO<br>- WFP
**Participating government & agencies**	- Ministry of Health (MoH)<br>- Ministry of Agriculture and Fisheries (MAF)<br>- Ministry of Education (MoE)<br>- Ministry of Commerce, Industry, and Environment (MCIE), initially named Ministry of Tourism, Commerce and Industry (MTCI)<br>- Ministry of Social Solidarity (MSS)
**Civil Society Organizations**	Alola Foundation, Pastoral da Crianca, Kailalo, Caritas, Moris Foun, Hamordade, Fundassaun Hadomi Progresu and YAACTS
**Dates**	13 November 2009 – 31 March 2013
**Regions of intervention**	Aileu, Baucau, Manatuto and Oecusse (4 out of 13 districts)
**Program evaluations**	Mid-term Evaluations in 2011 (Noij, 2011) and a final evaluation in November 2012 (Lenci, 2012).

[1]Main source: MDG-F, 2008.

MDG-F Joint Program: Objectives and strategy

The program's major goal was to improve and manage the health and nutrition status of a specific population, namely women of reproductive age and under-five children. This would ideally be realized by strengthening institutional capacity and service delivery mechanisms. An overview of specific goals

is given in table 2, as well as minor goals and the budget allocated to each. This strategy is designed to align with the United Nations Development Assistance Framework (UNDAF), with the MDG-Fund's own three pillars of development cooperation, namely to achieve the MDGs, to implement the principles of the Paris Declaration and to support UN reform, and lastly with Timor-Leste's national development strategies and policies, the so-called Comoro Declaration and Strategic Development Plan 2011-2030 (Gov. TL, 2010; Gov. TL, 2011). The MDG target concerned is MDG 1.8, stating that the prevalence of underweight children under five years should be halved between 1990 and 2015 (Lenci, 2012).

For demarcation reasons, the current study did not focus on evaluating whether the second and third expected outcomes were realized, as only small budgets and attention were allocated to their benefit. Also, for outcome number two, feasibility and adequacy of such evaluation would be limited, due to lacking information with regard to the theories that underlie the goals and concerning activities. In the MDG-F JP design, the target to increase school access and completion rates is assumed to be causally linked to the introduction to school gardens, which has not been substantiated with any reasoning (Lenci, 2012). Moreover, increasing children's access to education does not directly relate to food security and nutritional status, and is therefore of lesser relevance to this study's subject.

TABLE 2: OVERVIEW OF THE MDG-F JP'S STRATEGY

Expected output:	Budget:	Activities:
**Outcome 1: Improved health and nutritional status of pregnant and lactating women and under-five children in four selected districts**		
1.1 Strengthened capacities of the health system and local communities to increase availability of, access to and utilization of quality essential nutrition services at SISC posts, Health posts and Community health centers in four districts.	$850,326 (26%)	- Implementation and up-scaling of the Community Management of Acute Malnutrition Program, including training of staff, introduction of guidelines and protocols and provision of supplies and ready-to-use therapeutic food.
1.2 Increased demand for essential nutrition services by the families and communities, especially by the poor and vulnerable women and children in four districts.	$590,979 (18%)	- Establishing/supporting Mother Support Groups (MSGs). Educating these mothers in Infant and Young Child Feeding (IYCF) practices.
1.3 Increased production, availability and utilization of micronutrient-rich foods among women and children in four districts.	$1,394,985 (43%)	- Production pilot of *Timor Vita*, a fortified blended food for 2-5-years-old children.<br>- Distribution of corn soya blended powder.<br>- Micro-nutrient powder for 6-23-months-old children.<br>- Distribution of Vitamin A to 6-59-months-old children across 4 districts.<br>- Production of iodized salt by local farmers.<br>- Provision of training and supplies to farmers for the production of micronutrient rich food, through aquaculture and agriculture at household or community level.

**Outcome 2: 20% more children access, and 25% more children complete compulsory quality basic education in four selected districts.**		
2. Increased nutrition education in schools and communities through introduction of school gardens and consumption of nutritious foods, contributing to improved quality of meals provided by schools.	$259,739 (8%)	- Introduction of school gardens, including training, assistance and provision of resources.
**Outcome 3: Food security and nutrition surveillance system established and functioning at all sub-districts in four districts.**		
3.1 Strengthen capacities of Central and District Team to utilize food security information and Early Warning Systems (FSIEWS) at national, district and community levels	$135,000 (3.8%)	- Establishment of a FSIEWS at district and sub-district level by training of staff and developing a methodology.
3.2 Improved capacity of District Food Security and Disaster Management Committees to plan and support mitigation and response initiatives.	$40,000 (1.2%)	- Facilitating or convening meetings of relevant authorities using FSIEWS.

MDG-F Joint Program: Reported achievements

Various evaluation reports had been published prior to this study. A mid-term (Noij, 2011) and final evaluation (Lenci, 2012) had both been conducted by an independent international consultant, commissioned by UNICEF. A general paper on MDG-F joint programs around the globe had contributed some general achievements of the MDG-F JP in Timor-Leste (United Nations, 2013). Ultimately, a case study evaluation was conducted, which mainly focused on the MDG-F JP's process (Fernandes, 2012). Reasons for conducting the mid-term and final evaluation were that the quality of the MDG-F JP design, the implementation process and the final results needed to be accounted for institutionally. Additionally, future designing of programs would benefit from identified barriers and lessons.

Primarily, relevance, effectiveness, efficiency, ownership and sustainability of the MDG-F JP were each assessed in Lenci's evaluation (2012). An overview of those outcomes will be discussed here, supplemented by reported achievements by various evaluation reports. The first criterium, *relevance*, has entirely been met by the integrated approach of the MDG-F JP to tackle a key problem in Timor-Leste. *Effectiveness* was constricted by the suboptimal synergy between activities related to nutrition security on the one hand, and food security on the other hand. Operational focus was largely on nutrition security, creating an incomplete integrated approach. Further constraints for sustainable outcomes were found in design, implementation and institutional cooperation (Lenci, 2012). According to Lenci, no nutritional or health status data was available for a quantitative assessment of actual impact of the food and nutrition program on the target population (2012). Qualitative outcomes from interviews on the other hand, did reveal that access to nutrition services had increased. Especially reaching remote communities has been a challenge of which results remain unknown. The total number of 139 established Farmers Groups (FG) appeared to increase food and income generation (Lenci, 2012; MDG-F, 2008). The fourteen Mother Support Groups (MSGs) were reported to boost awareness and **child feeding and/or care capacities**. Additionally, improved **home-gardening** knowledge and practices fortuitously resulted in the **increased generation of household income** by women and their families (Lenci, 2012; United Nations, 2013). Constraints to effectiveness were, however, that the intervention providing food supplementation to mothers were targeted at other districts than the MSGs intervention, creating a blockage in the integration process. This joint program did *not* include targeted strategies for staple crop production, legume production or biofortification. Also, no specific design aspects were included to increase household income (United Nations, 2013).

The food production activities in relation to output 1.3 have unintentionally led to local market sale of produce, which is considered positive (Lenci, 2012). In practice, **strengthening institutional capacity and service delivery** by means of this joint program has partially effectively been done through constructing multi-level stakeholder partnerships, in the public- as well as the private sector. For example, public-private partnerships were involved in the introduction of fortified food supplements at the local level (United Nations, 2013). Strategies to **improve access to markets** were stated to have led to **diversification of diet composition**, but details are lacking on the actual strategies (United Nations, 2013).

The concept of *efficiency* was difficult to assess, since stakeholder involvement is inevitably time and energy consuming, paying off later on or in a less transparent way. Altogether, efficiency was reported to be achieved to a large extent. Nonetheless, nutrition supplies were not always distributed adequately and recruiting MDG-F JP staff went suboptimal. Also, stakeholders were often not familiar with the methods and formats that were used, which often constituted constraints to efficient cooperation and action. *Ownership* of the MDG-F JP by local parties increased over time. The implementation process was initialized shortly after Timor-Leste's crisis situation, and was therefore mostly governed by UN agencies at first. By taking local capacities and needs into account, crop selection was conducted to optimize **local production of nutrient-dense foods**. This was achieved through educational efforts (United Nations, 2013). Furthermore, the MDG-F joint program in Timor-Leste focused on strengthening the country's **food security and nutrition surveillance system**. This has been done through capacity building of professionals in Governmental institutions on both the central and field level in each of the thirteen districts. These people improved their expertise in collecting, compiling and analyzing data to develop a Food Security Information and Early Warning System (FSIEWS) (United Nations, 2013). Budget allocation to the Ministries of Health and Agriculture are increasing as a result of **increased political recognition and commitment** to the JP's issues. However, relative to the overall government budget, funds are not considered to be significantly increasing. This also relates to the sustainability aspect of Lenci's assessment. *Sustainability* is increased when interventions are aimed at developing capacities for further maintenance and innovation, as well as at increasing income generation, thereby creating an economic incentive. This is for example the case with home gardens and livestock breeding. Nonetheless, technical assistance and funds are especially crucial in the first phase of implementation in order to establish sustainability after the program has ended. Lastly, it is very important to increase production and reduce costs of interventions, and to thereby decrease dependency on humanitarian assistance, for example for production of Timor Vita fortified and supplementary feeding.

Interestingly, the national evaluation team's report concluded with the recommendation to make sure that stakeholders are involved, and that leadership by the government and other associated institutions is supported as much as needed (Fernandes, 2012). But not only that, an integrated approach was reported to be essential at the local level, which was partially to be achieved through communication, campaigns, coordination (multi-sectoral) and increased transparency of money flows. Finally, it was stated to be very challenging to establish a sustainable system with adequate interventions and guaranteed allocation and management of resources over time (Fernandes, 2012).

MDG-F Joint Program: Additional MDG-F support

In 2009, the MDG Fund selected eight countries, which would receive support for Advocacy and Communication (A&C) and Monitoring and Evaluation (M&E) strategies, in addition to the joint programs already running there (MDG-F, 2009a; Fernandes, 2012). A budget of $210,000 across three years was allocated to the A&C strategy (MDG-F, 2009a) and $300,000 to the M&E strategy (MDG-F, 2009b). By means of the first strategy, an increase of specific stakeholders' understanding, awareness and support for the MDGs had to be established, whereas the latter strategy was aimed at making

sure that participatory case study evaluations were conducted after implementation of the joint programs (MDG-F, 2009b).

A major finding of the final evaluation was that the MDG-F JP did indeed increase recognition and prioritization of food and nutrition security issues by the government of Timor-Leste, through these advocacy and communication efforts (Lenci, 2012). The evaluation showed that the A&C strategy increased responsiveness in the areas of nutrition and food security 'somewhat' (Fernandes, 2012). It also 'somewhat' increased engagement with and appreciation of the MDG among Timorese citizens and strengthened 'somewhat' national ownership and improved considerably coordination among all partners (Fernandes, 2012). The M&E initiatives on the other hand, were perceived by partners as contributing 'to a great extent' to individual and organizational learning, to improving capacities and to the overall evidence-based quality of the joint programs. They were also stated to strengthen national ownership significantly (Fernandes, 2012).

MDG-F Joint Program: Evaluation gaps

The previously conducted MDG-F JP evaluations focused on improvement of national ownership and coordination mechanisms, more than on the achievement of food security goals (Lenci, 2012). Other constraints were that they were mainly substantiated by qualitative analyses (Lenci, 2012). Quantity of food produced and actual service coverage within the target population were reported not to have been monitored, and were therefore not included in the evaluations (Lenci, 2012). Improved capacities, cooperation or communication for example, were not further specified (Fernandes, 2012). Therefore, remaining evaluation gaps made this extensive study particularly relevant. At the same time, the described evaluations offered insights into national ownership and related concepts as relevant factors promoting or hampering success of a food security initiative.

References

Fernandes, R. (2012). Timor-Leste MDG-F Case Study Evaluation. Final evaluation report. Accessed on 8 Feb. 2021. Retrieved from:
http://www.mdgfund.org/sites/default/files/Timor%20Leste_Country%20Final%20Evaluation.pdf

Government of Timor-Leste. (2010). Comoro Declaration "Putting an End to Hunger and Malnutrition", Dili, Timor-Leste, 18 October 2010. Government of Democratic Republic of Timor-Leste, Dili, 2010. 10 pp.

Government of Timor-Leste. (2011). Timor-Leste Strategic Development Plan 2011-2030.213 pp. Retrieved from:
http://www.tls.searo.who.int/LinkFiles/Home_NATIONAL_STRATEGIC_DEVELOPMENT_PLAN_2011-2030.pdf.

Lenci S. (2012). MDG-F JP Sustainable Food and Nutrition Security in Timor-Leste Final Evaluation Report. MDG Fund. Retrieved from:
http://www.mdgfund.org/sites/default/files/Timor%20Leste%20-%20Nutrition%20-%20Final%20Evaluation%20Report.pdf.

MDG-F. (2008). Timor-Leste. Joint Programmes Fact Sheet. Accessed on 6 March 2021. Retrieved from: http://mdgfund.org/country/timorleste.

MDG-F. (2009a). Timor-Leste Advocacy Plan. Timor-Leste's MDG-F Advocacy & Partnerships. Accessed on 2021 Feb. 12. Retrieved from:
http://www.mdgfund.org/sites/all/themes/custom/undp_2/docs/Timor-Leste%20Advocacy%20Plan.pdf.

MDG-F. (2009b). MDG-F: Monitoring and Evaluation. Action Plan of Timor Leste. Accessed on 2021 Feb. 12. Retrieved from: http://www.mdgfund.org/sites/all/themes/custom/undp_2/docs/Timor-Leste%20M&E%20Plan.pdf.

Noij, F. (2011). Joint program promoting sustainable food and nutrition security in Timor-Leste: Mid-term evaluation—final report.

United Nations. (2013) JOINT PROGRAMMES FOR FOOD SECURITY AND NUTRITION. A qualitative review of agricultural programming for nutrition among the Millennium Development Goals Achievement Fund joint programmes. Accessed on 12 Feb. 2021. Retrieved from: https://www.sdgfund.org/joint-programmes-food-and-nutrition-security
