# Supplementary material for: Analyzing the impact of an MDG-Fund program on childhood malnutrition in Timor-Leste
Source: J Health Popul Nutr. 2024 Apr 4;43:46. doi: 10.1186/s41043-024-00539-x (PMC10993443; doi:10.1186/s41043-024-00539-x)

Article: *Analyzing the impact of an MDG-Fund program on childhood malnutrition in Timor-Leste;*
Journal: *Environment, Development and Sustainability;*
Authors: *H.D. van der Spek, MSc. (lindavdspek@live.nl) and Dr. B.G.J.S. Sonneveld (b.g.j.s.sonneveld@vu.nl).*

Online Resource 3: Flow chart of the study population

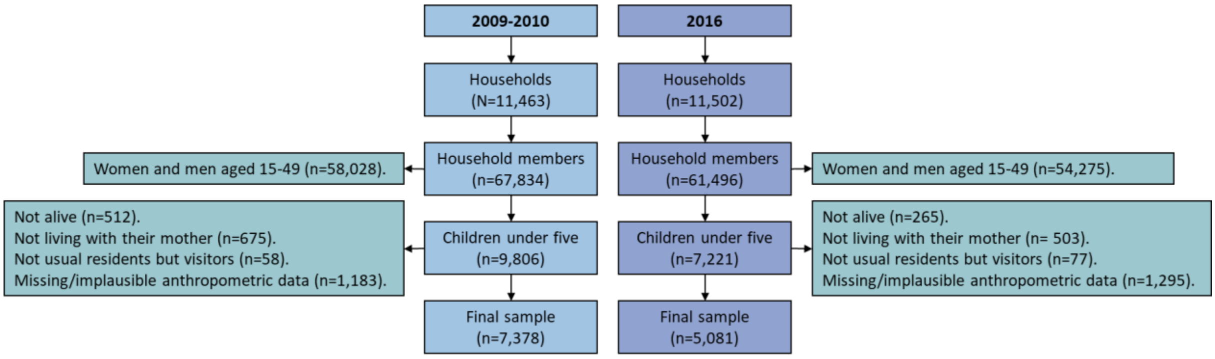

Supplement: Supplementary file 3 — Additional file 3. Flow chart of the study population. [file 41043_2024_539_MOESM3_ESM.pdf]
