# Supplementary material for: Analyzing the impact of an MDG-Fund program on childhood malnutrition in Timor-Leste
Source: J Health Popul Nutr. 2024 Apr 4;43:46. doi: 10.1186/s41043-024-00539-x (PMC10993443; doi:10.1186/s41043-024-00539-x)
Supplement: Supplementary file 4 — Additional file 4. Characteristics of children in the sample. [file 41043_2024_539_MOESM4_ESM.pdf]

Article: *Analyzing the impact of an MDG-Fund program on childhood malnutrition in Timor-Leste;*
Journal: *Environment, Development and Sustainability;*
Authors: *H.D. van der Spek, MSc. (lindavdspek@live.nl) and Dr. B.G.J.S. Sonneveld (b.g.j.s.sonneveld@vu.nl).*

Online Resource 4: Characteristics of children in the sample

		2009-2010		2016	
Characteristic		No.	Est.	No.	Est.
Children in the sample		7378		5081	
Age of child	0	1445	19,59%	1019	20,06%
	1	1413	19.15%	1037	20,41%
	2	1524	20.66%	990	19,48%
	3	1577	21.37%	1053	20,72%
	4	1419	19.23%	982	19,33%
Child's anemia level	Not anemic	1207	60,08%	801	60,77%
	Mild	494	24.59%	347	26,33%
	Moderate	292	14.53%	166	12,59%
	Severe	16	0.80%	4	0,30%
Male sex		3710	50.28%	2583	50,84%
Minimum Dietary Diversity		1487	23.37%	488	15,09%
Size of child at birth	Smaller	1047	14,46%	360	8,81%
	Average	4379	60.47%	2867	70,15%
	Larger	1816	25.08%	860	21,04%
**Household characteristics:**					
Female-headed household		478	6.48%	540	10,63%
Number of household members, mean (SD)		7378	7.42 (2.61)		7,11 (2,86)
Improved source of drinking water		4302	59.13%	961	26,39%
Improved sanitation facility		3500	47.74%	1581	33,31%
Wealth index factor score, mean (SD)		7378	-.07 (.90)	5081	0.07 (0.97)
Wealth index	Poorest	1726	23,39%	986	19,41%
	Poorer	1559	21.13%	1078	21,22%
	Middle	1571	21.29%	1047	20,61%
	Richer	1456	19.73%	1100	21,65%
	Richest	1066	14.45%	870	17,12%
Owning land usable for agriculture		6508	95.54%	4115	87,78%
Hectares of agricultural land, median (IQR)		3118	1 (1 to 2)	2708	10 (9 to 20)
Owns livestock, herds or farm animals		6450	94.69%	4264	90,96%
Rural area of residence		5796	78.56%	3604	70,93%
Living in an MDGF-JP district		2489	33.74%	1738	34,21%
Household size	0-5	1720	23,31%	1529	30,09%
	6-10	4881	66.16%	3035	59,73%
	>10	777	10.53%	517	10,18%
Hectares of agricultural land	No land	134	4,30%	656	24,22%
	1 ha	1460	46.82%	10	0,37%
	2 ha	1013	32.49%		
	>= 3 ha	511	16.39%	2042	75,41%
District of residence	Aileu	954	12,93%	795	15,65%

	Ainaro	1049	14.22%	509	10,02%
	Baucau	440	5.96%	324	6,38%
	Bobonaro	427	5.79%	391	7,70%
	Cova Lima	377	5.11%	284	5,59%
	Dili	567	7.69%	520	10,23%
	Ermera	561	7.60%	301	5,92%
	Liquica	493	6.68%	360	7,09%
	Lautem	574	7.78%	320	6,30%
	Manufahi	420	5.69%	351	6,91%
	Manatuto	518	7.02%	322	6,34%
	Oecusse	577	7.82%	297	5,85%
	Viqueque	421	5.71%	307	6,04%
Altitude	=< 800	1707	23,14%	1278	25,15%
	800-1000	1038	14,07%	889	17,50%
	1000-1200	2405	32,60%	2102	41,37%
	> 1200	2228	30,20%	812	15,98%
Mean Vegetation Health Index (MVHI)	First quantile	1890	25,62%	1167	22,97%
	Second quantile	1447	19,61%	1155	22,73%
	Third quantile	1244	16,86%	999	19,66%
	Fourth quantile	2797	37,91%	1760	34,64%
Population density	=< 50	1328	18,00%	969	19,07%
	50-100	2396	32,47%	1365	26,86%
	100-500	2885	39,10%	2104	41,41%
	> 500	769	10,42%	643	12,65%
Crop cover	=< 1%	1991	26,99%	1258	24,76%
	1-5%	1639	22,21%	1192	23,46%
	5-10%	2171	29,43%	1371	26,98%
	>10%	1577	21,37%	1260	24,80%
Soil suitability	Marginal	864	11,71%	696	13,70%
	Moderate	1645	22,30%	309	6,08%
	Medium	4305	58,35%	3705	72,92%
	Good	564	7,64%	371	7,30%
Slope	Marginal	2605	35,31%	2053	40,41%
	Moderate	3630	49,20%	2183	42,96%
	Medium	1143	15,49%	845	16,63%
	Cow or bull	2334	34.26%	1693	41,05%
	Horse, donkey or mule	2474	36.32%	849	18,11%
	Goat or sheep	2921	42.88%	1747	37,27%
	Chicken	5654	83.00%	3671	78,31%
**Mother's characteristics:**					
BMI (W/H2), mean (SD)		7280	20.49 (2.99)	5031	21,20 (3,33)
BMI	Underweight	1316	17,84%	832	16,37%
	Normal weight	5627	76.27%	3634	71,52%
	Overweight	435	5.90%	615	12,10%
Not anemic		1889	77.35%	1235	73,56%

Smoking		393	5.33%	254	4,99%
Using contraception		1766	23.94%	1480	29,13%
Uneducated		2562	34,72%	1258	24,76%
Currently working		3064	41.53%	1901	37,41%
Occupation	Not working	4276	58,03%	3045	59,93%
	Agric-(self) employed	2038	27.66%	657	12,94%
	Other	1055	14.32%	1375	27,08%
Uneducated partner		2116	28,70%	1128	22,70%
Partner's occupation	Not working			963	19,38%
	Agric-(self) employed	4600	62.66%	1398	32,62%
	Other	2741	37.33%	1925	45,54%
Mother’s anemia level	Severe	9	0,37%	4	0,24%
	Moderate	103	4.22%	98	5,84%
	Mild	441	18.06%	342	20,37%
	Not anemic	1889	77.35%	1235	73,56%
Mother’s education level	None	2562	34,72%	1258	24,76%
	Incomplete primary	1163	15.76%	508	10,00%
	Primary	954	12.93%	439	8,64%
	Incomplete secondary	1548	20.98%	1189	23,40%
	Complete secondary	1044	14.15%	1284	25,27%
	Higher	107	1.45%	403	7,93%
Partner's education level	None	2116	28,70%	1128	22,70%
	Primary	2051	27.81%	1048	21,09%
	Secondary	2869	38.91%	2140	43,06%
	Higher	331	4.49%	649	13,06%
