# Supplementary material for: Analyzing the impact of an MDG-Fund program on childhood malnutrition in Timor-Leste
Source: J Health Popul Nutr. 2024 Apr 4;43:46. doi: 10.1186/s41043-024-00539-x (PMC10993443; doi:10.1186/s41043-024-00539-x)
Supplement: Supplementary file 8 — Additional file 8. Model selection. [file 41043_2024_539_MOESM8_ESM.pdf]

Article: *Analyzing the impact of an MDG-Fund program on childhood malnutrition in Timor-Leste;*
Journal: *Environment, Development and Sustainability;*
Authors: *H.D. van der Spek, MSc. (lindavdspek@live.nl) and Dr. B.G.J.S. Sonneveld (b.g.j.s.sonneveld@vu.nl).*

Online Resource 8: Model selection

a. Effects selected[1] in at least 20% of the samples for HAZ-scores

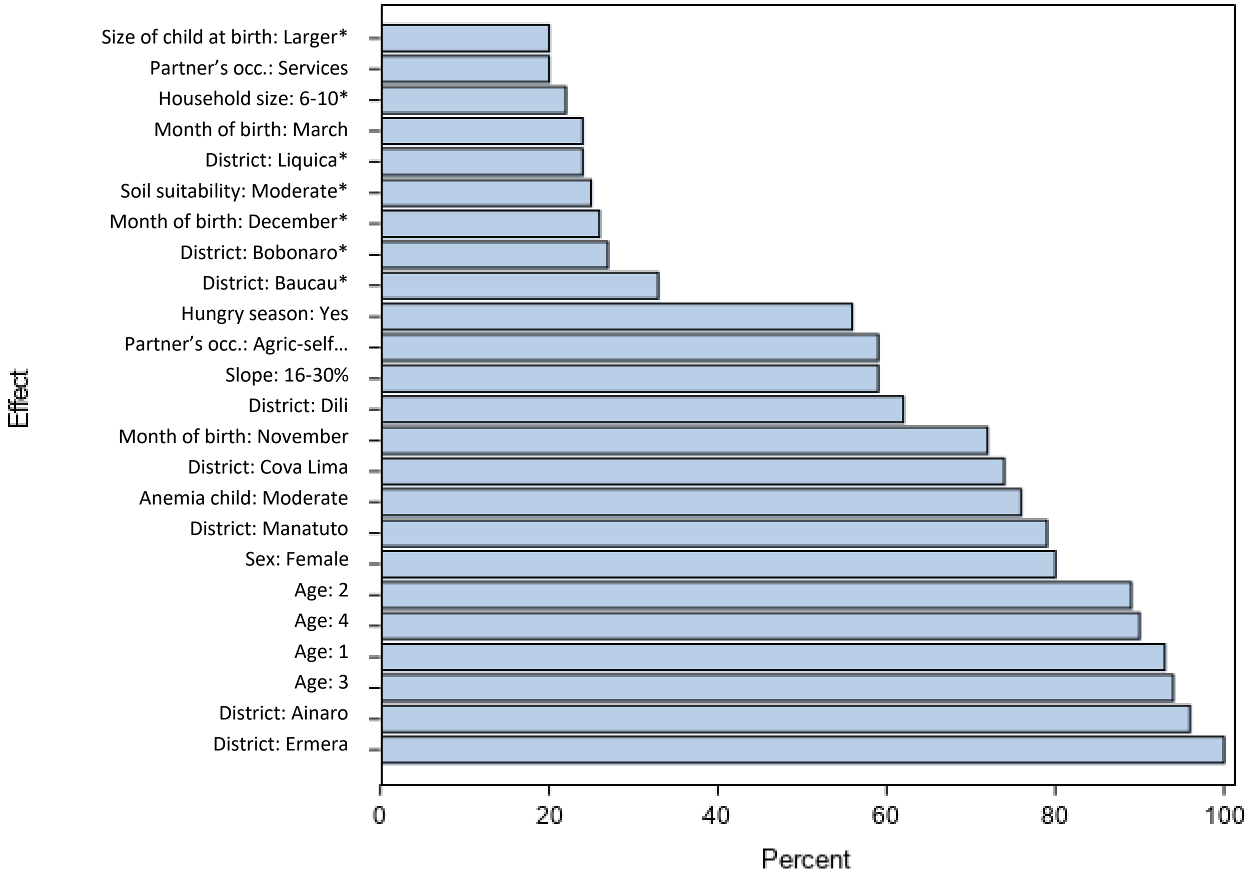

[1]Effects with an asterisk were included in the main model. Effects without an asterisk were excluded based on the p-value criterium of 0.05.

b. Effects selected[1] in at least 20% of the samples for WAZ-scores

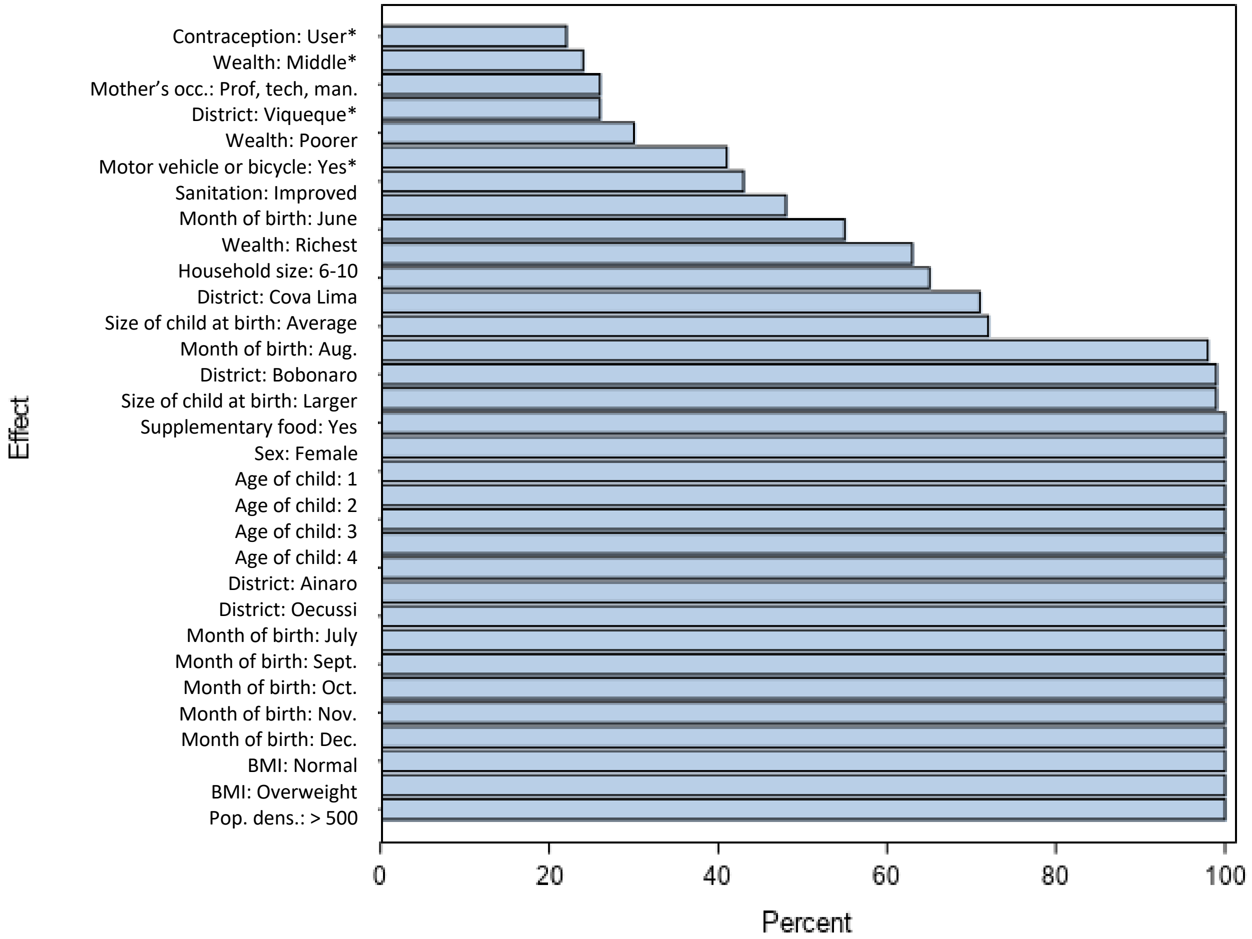

[1]Effects with an asterisk were included in the main model. Effects without an asterisk were excluded based on the p-value criterium of 0.05.

c. Effects selected[1] in at least 20% of the samples for WHZ-scores

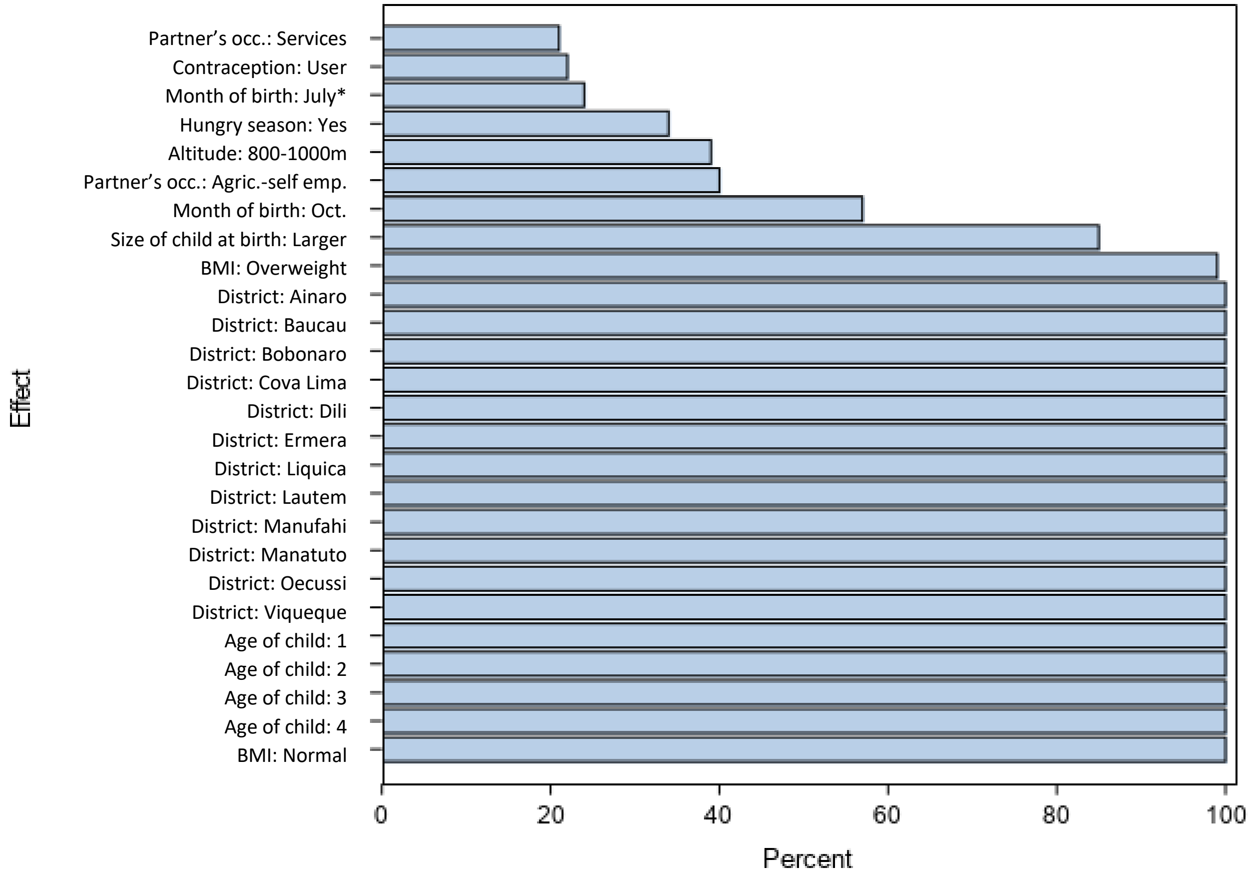

[1]Effects with an asterisk were included in the main model. Effects without an asterisk were excluded based on the p-value criterium of 0.05.
